# HDAC4 stimulates MRTF-A expression and drives fibrogenesis in hepatic stellate cells by targeting miR-206

**DOI:** 10.18632/oncotarget.17739

**Published:** 2017-05-10

**Authors:** Xinrui Han, Chenzhi Hao, Luyang Li, Jianfei Li, Mingming Fang, Yuanlin Zheng, Jun Lu, Ping Li, Yong Xu

**Affiliations:** ^1^ Key Laboratory for Biotechnology on Medicinal Plants of Jiangsu Province, School of Life Science, Jiangsu Normal University, Xuzhou, China; ^2^ Department of Pathophysiology, Key Laboratory of Cardiovascular Disease and Molecular Intervention, Nanjing Medical University, Nanjing, China; ^3^ Department of Gastroenterology, 2nd Affiliated Hospital to Nanjing Medical University, Nanjing, China

**Keywords:** HDAC4, hepatic stellate cell, liver fibrosis, MRTF-A, miRNA

## Abstract

Activation of hepatic stellate cells (HSCs) is a hallmark event during liver fibrogenesis. We have previously shown that the transcriptional modulator MRTF-A contributes to liver fibrosis by programming epigenetic activation of HSCs. In the present study we investigated the mechanism whereby MRTF-A expression is regulated in this process. We report here that MRTF-A protein levels, but not mRNA levels, were up-regulated *in vivo* in the livers of mice induced to develop hepatic fibrosis. Pro-fibrogenic stimuli (TGF-β and PDGF-BB) also activated MRTF-A expression post-transcriptionally *in vitro* in cultured HSCs. miR-206 bound to the 3′-UTR of MRTF-A presumably to inhibit translation. miR-206 levels were down-regulated in response to pro-fibrogenic stimuli *in vivo* and *in vitro* allowing MRTF-A proteins to accumulate. Mechanistically, histone deacetylase 4 (HDAC4) was induced by pro-fibrogenic stimuli and recruited to the miR-206 promoter to repress miR-206 transcription. HDAC4 stimulated MRTF-A expression and drove fibrogenesis in HSCs in a miR-206 dependent manner. Therefore, our data reveal an HDAC4-miR-206-MRTF-A axis that can play a potentially important role in HSC activation and liver fibrosis.

## INTRODUCTION

Liver fibrosis is perceived as a host defense mechanism that aims to facilitate wound healing and restoration of hepatic function. Un-controlled liver fibrosis, however, can lead to debilitating conditions such as cirrhosis and hepatocellular carcinoma posing a severe health risk [1]. The production of extracellular matrix (ECM) proteins, including collagen type I and collagen type III, is significantly elevated during liver fibrosis. Hepatic stellate cells (HSCs) represent a major source of ECM synthesis contributing to liver fibrogenesis [2]. Generally functioning as a reservoir for certain lipids (e.g., vitamin A) when in quiescent state, HSCs can assume a myofibroblast-like phenotype, or “activated”, and accelerate the production of ECM proteins once exposed to a number of pro-fibrogenic stimuli including transforming growth factor (TGF-β) and platelet derived growth factor (PDGF-BB) [3]. A thorough understanding of the HSC pathobiology could lead to breakthroughs in the development of novel strategies against malicious liver fibrosis.

Previously, our investigation has led to the discovery of myocardin-related transcription factor A (MRTF-A) as a key modulator of HSC activation and liver fibrosis [4]. MRTF-A contributes to this process primarily by epigenetically regulating the TGF-β signaling pathway [5, 6]. In the meantime, we inadvertently found that when primary mouse hepatic stellate cells underwent spontaneous activation *in vitro* MRTF-A proteins levels, but not mRNA levels, were up-regulated suggestive of a post-transcriptional mechanism with the possible involvement of microRNAs.

MicroRNAs, or miRNAs, refer to a group of non-coding RNAs ~22nt in size. miRNAs play a wide range of roles regulating life activities. Recent studies have implicated miRNAs as both a biomarker and a target for treatment in hepatocellular carcinoma, whose development often precedes dysregulated liver fibrosis [7]. Of key significance, a profiling study has found that levels of multiple miRNAs were correlated with, either positively or inversely, the development of liver fibrosis in mice [8], indicating that miRNAs may directly participate in the regulation of HSC activation. Here we identify miRNA-206 as a regulator of MRTF-A expression in HSCs and provide evidence to show that HDAC4 epigenetically controls the transcription of miR-206 to stimulate MRTF-A expression and drive fibrogenesis. Therefore, this HDAC4-miR-206-MRTF-A axis may serve as a potential target for developing novel interventional strategies to prevent malicious liver fibrosis.

## RESULTS

### Pro-fibrogenic stimuli activates MRTF-A expression post-transcriptionally

**Figure 1:**
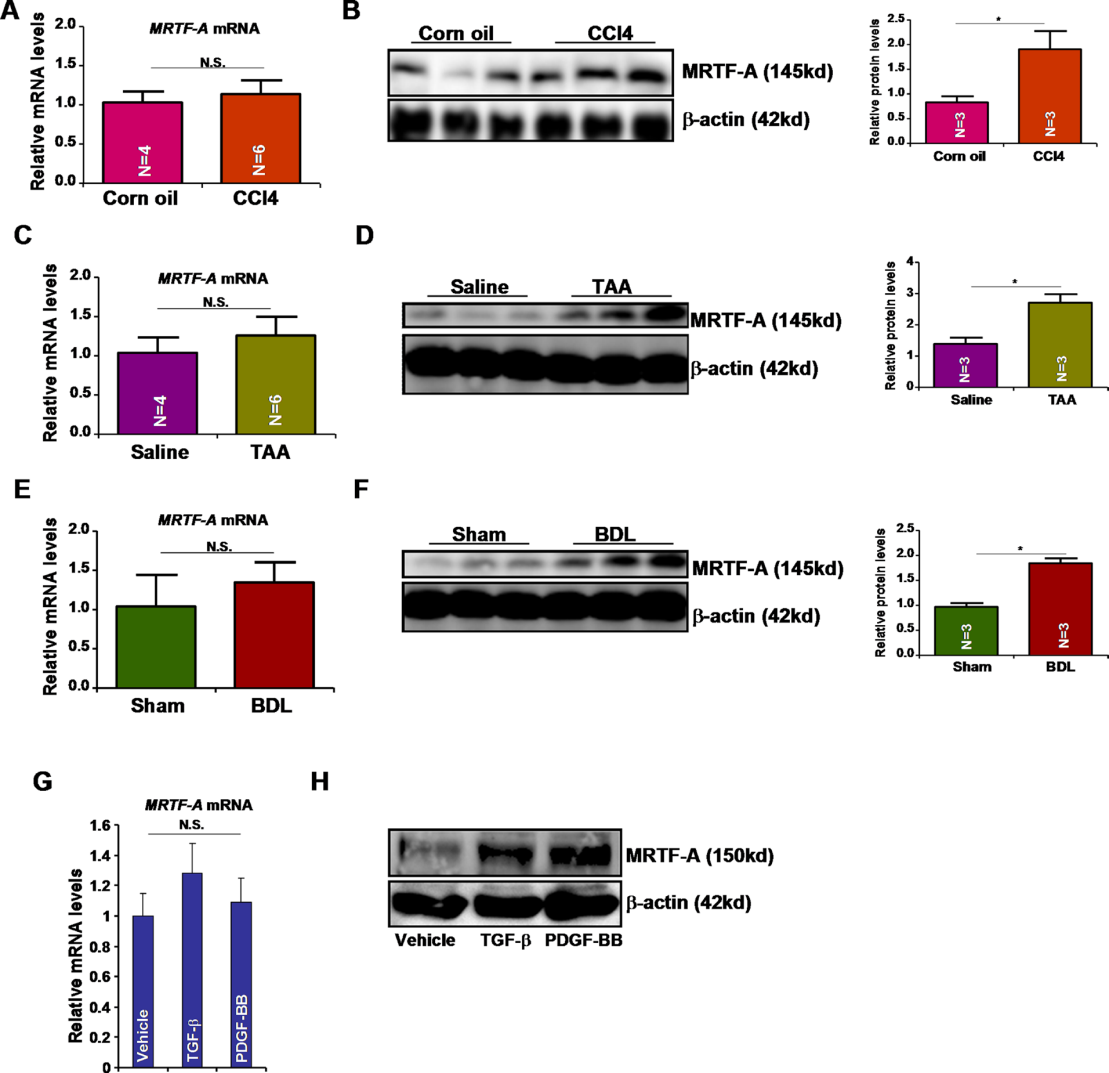
Pro-fibrogenic stimuli activates MRTF-A expression post-transcriptionally (**A, B**) Liver fibrosis was induced in C57/BL6 mice by CCl4 injection. Hepatic MRTF-A expression was evaluated by qPCR (A) and Western (B). (**C, D**) Liver fibrosis was induced in C57/BL6 mice by TAA injection. Hepatic MRTF-A expression was evaluated by qPCR (C) and Western (D). (**E, F**) Liver fibrosis was induced in C57/BL6 mice by bile duct ligation (BDL). Hepatic MRTF-A expression was evaluated by qPCR (E) and Western (F). (**G, H**) HSC-T6 cells were treated with TGF-β or PDGF-BB for 24 hours. MRTF-A expression was evaluated by qPCR (G) and Western (H).

Previously, we have shown that MRTF-A protein levels were up-regulated in activated primary mouse hepatic stellate cells compared to quiescent cells without a concomitant increase in mRNA expression indicative of a post-transcriptionally regulation [4]. We then verified this observation in mice. In a classic model of liver fibrosis in which C57/BL6 mice were given daily peritoneal injection of CCl4 for a week, we found that whereas there was a significant increase in MRTF-A protein expression message levels of MRTF-A were not altered (Figure 1A, 1B). In two separate mouse models of liver fibrosis, one induced by peritoneal injection of thioacetamide (TAA) and the other by bile duct ligation (BDL), we were able to further validate that MRTF-A protein levels, rather than mRNA levels, were correlated with hepatic fibrogenesis (Figure 1C–1F). In addition, two major pro-fibrogenic growth factors, transforming growth factor (TGF-β) and platelet derived growth factor (PDGF-BB), markedly stimulated protein expression of MRTR-A without impacting message levels in cultured rat hepatic stellate cells (HSC-T6, Figure 1G and 1H). Therefore, we conclude that pro-fibrogenic stimuli activate MRTF-A expression post-transcriptionally.

### miR-206 regulates MRTF-A expression

**Figure 2:**
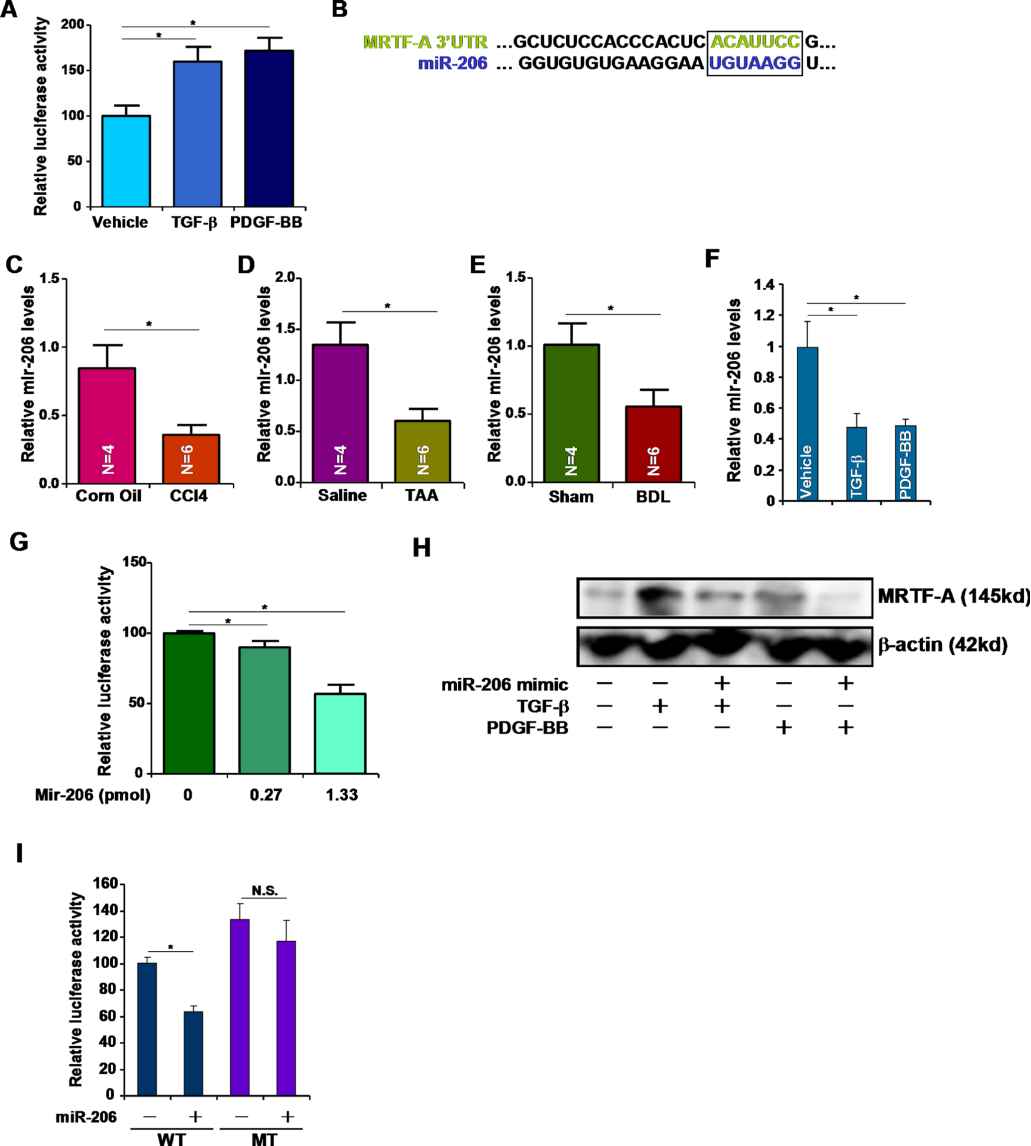
miR-206 regulates MRTF-A expression (**A**) A luciferase construct harboring the 3′-UTR of MRTF-A was transfected into HSC-T6 cells followed by with TGF-β or PDGF-BB for 24 hours. Luciferase activities were normalized by both protein concentration and GFP fluorescence. (**B**) Alignment of the 3′-UTR of the MRTF-A gene and miR-206. Matching sequences are highlighted and boxed. (**C**–**E**) Hepatic expression of miR-206 was examined by qPCR in (C) CCl4-, (D) TAA-, and (E) BDL-induced liver fibrosis in mice. (**F**) HSC-T6 cells were treated with TGF-β or PDGF-BB for 24 hours. Expression of miR-206 was examined by qPCR. (**G**) A luciferase construct harboring the 3′-UTR of MRTF-A was transfected into HSC-T6 cells with increasing doses of miR-206 mimic. Luciferase activities were normalized by both protein concentration and GFP fluorescence. (**H**) A miR-206 mimic was transfected into HSC-T6 cells followed by treated with TGF-β or PDGF-BB. MRTF-A expression was examined by Western. (**I**) A wild type (WT) or mutated (MT) MRTF-A reporter construct was transfected into HSC-T6 cells with or without miR-206. Luciferase activities were normalized by both protein concentration and GFP fluorescence.

To confirm that TGF-β and PDGF regulate MRTF-A expression post-transcriptionally, we cloned approximately 2kb of the 3′ un-translated region (UTR) of the MRTF-A mRNA into the pGL4 vector. Reporter assays performed in HSC-T6 cells showed that both TGF-β and PDGF up-regulated the activity of the MRTF-A 3′-UTR (Figure 2A). We then hypothesized that some MRTF-A targeting miRNA(s) might be down-regulated during liver fibrogenesis and thus allow MRTF-A messages to accumulate and be translated. Roderburg et al have reported a panel of miRNAs whose levels were decreased in the livers of Balb/c mice receiving CCl4 injection [8]. Among those miRNAs, we were able to match miR-206 with the 3′ UTR of MRTF-A (Figure 2B). Quantitative PCR analyses confirmed that miRNA-206 levels were reduced in the livers in mice induced to develop hepatic fibrosis by receiving CCl4 injection (Figure 2C), TAA injection (Figure 2D), or undergoing BDL (Figure 2E). Additionally, TGF-β and PDGF repressed miR-206 expression in HSC-T6 cells (Figure 2F). Two lines of evidence supported the connection between miR-206 and MRTF-A. First, over-expression of miR-206 (mimic) down-regulated the activities of the MRTF-A 3′-UTR reporter in a dose-dependent manner (Figure 2G). Second, the miR-206 mimic directly down-regulated endogenous MRTF-A protein levels in HSC-T6 cells (Figure 2H). When the putative miR-206 recognition site was mutated, the MRTF-A reporter lost its responsiveness to the miR-206 mimic (Figure 2I). Taken together, these data suggest that miR-206 may play a role in the regulation of MRTF-A expression during liver fibrosis.

### HDAC4 represses miR-206 transcription

**Figure 3:**
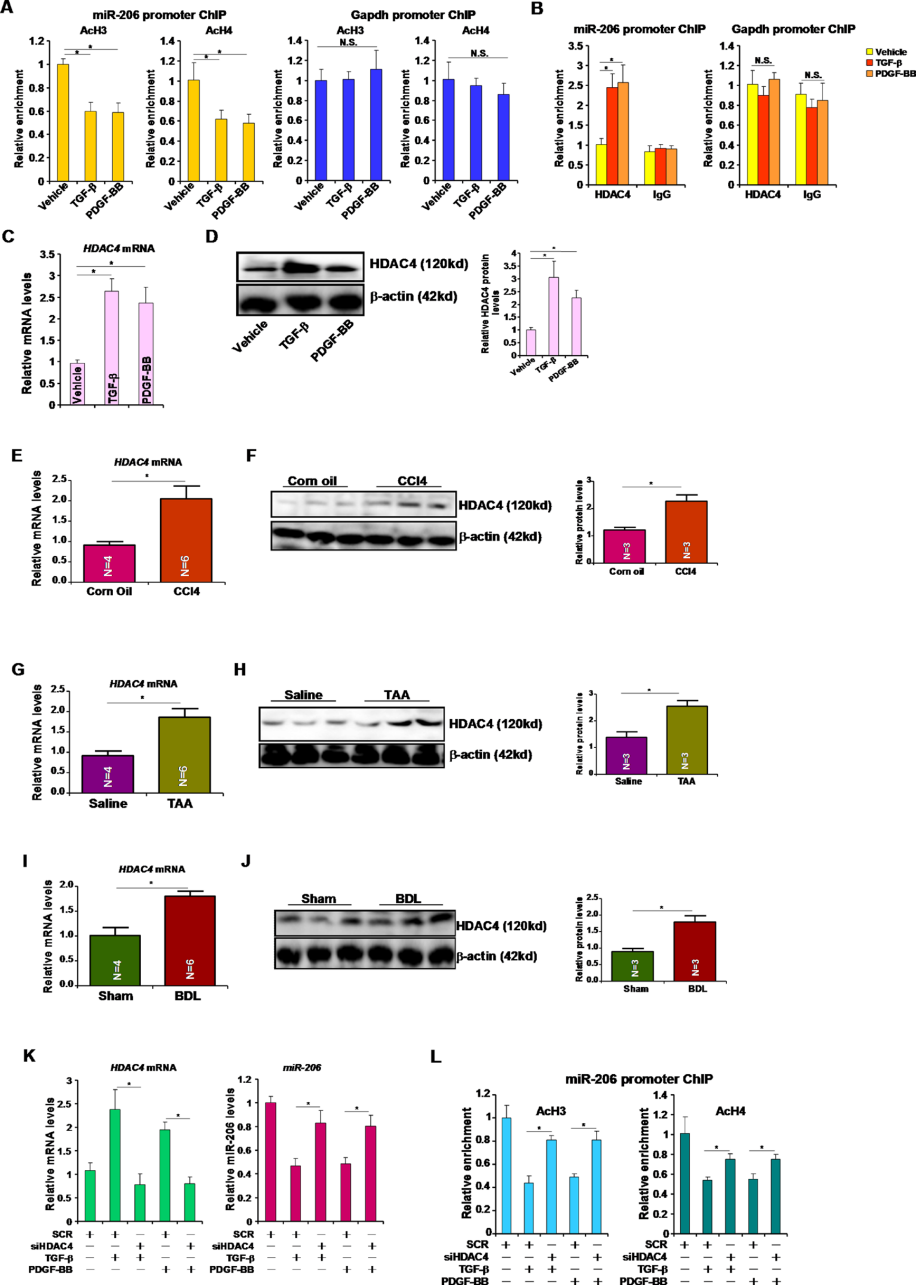
HDAC4 represses miR-206 transcription (**A, B**) HSC-T6 cells were treated with TGF-β or PDGF-BB for 24 hours. ChIP assays were performed with indicated antibodies. (**C, D**) HSC-T6 cells were treated with TGF-β or PDGF-BB for 24 hours. HDAC4 expression was examined by qPCR (C) and Western (D). (**E, F**) Hepatic expression of HDAC4 was examined by qPCR (E) and Western (F) in CCl4-induced liver fibrosis model. (**G, H**) Hepatic expression of HDAC4 was examined by qPCR (G) and Western (H) in TAA-induced liver fibrosis model. (**I, J**) Hepatic expression of HDAC4 was examined by qPCR (G) and Western (H) in BDL-induced liver fibrosis model. (**K, L**) HSC-T6 cells were transfected with siRNA targeting HDAC4 or scrambled siRNA followed by treatment with TGF-β or PDGF-BB for 24 hours. Expression levels of HDAC4 and miR-206 were examined by qPCR (K). ChIP assays were performed with indicated antibodies (L).

Next, we tackled the mechanism whereby miRNA-206 expression might be repressed by pro-fibrogenic stimuli. ChIP assays revealed that following treatment with TGF-β or PDGF, acetylated histone H3 (AcH3) and acetylated histone H4 (AcH4) were down-regulated surrounding the miR-206 proximal promoter (Figure 3A). We did not detect any changes in AcH3 or AcH4 surrounding the Gapdh promoter (Figure 3A). Several independent investigations have demonstrated that the histone deacetylase HDAC4 is involved in regulating miR-206 in different cells [9, 10]. ChIP assays showed that in response to TGF-β or PDGF treatment, occupancies of HDAC4 on the miR-206 promoter, but not the Gapdh promoter, were augmented (Figure 3B). HDAC4 expression was up-regulated in HSC-T6 cells following TGF-β or PDGF stimulation (Figure 3C, 3D) and in the livers of mice induced to develop hepatic fibrosis by receiving CCl4 injection (Figure 3E, 3F), TAA injection (Figure 3G, 3H), or undergoing BDL (Figure 3I, 3J), suggesting that HDAC4 levels may potentially serve as a marker for liver fibrogenesis. To prove that HDAC4 was indeed responsible for miR-206 repression in hepatic stellate cells, we used small interfering RNA (siRNA) to deplete endogenous HDAC4. HDAC4 knockdown abrogated repression of miR-206 induced by TGF-β or PDGF (Figure 3K). In the meantime, HDAC4 depletion also normalized histone H3 acetylation and H4 acetylation on the miR-206 promoter (Figure 3L). Collectively, these data illustrate an HDAC4-dependent pathway that mediates TGF-β/PDGF-induced repression of miR-206 in hepatic stellate cells.

### HDAC4 regulates fibrogenesis by targeting miR-206

**Figure 4:**
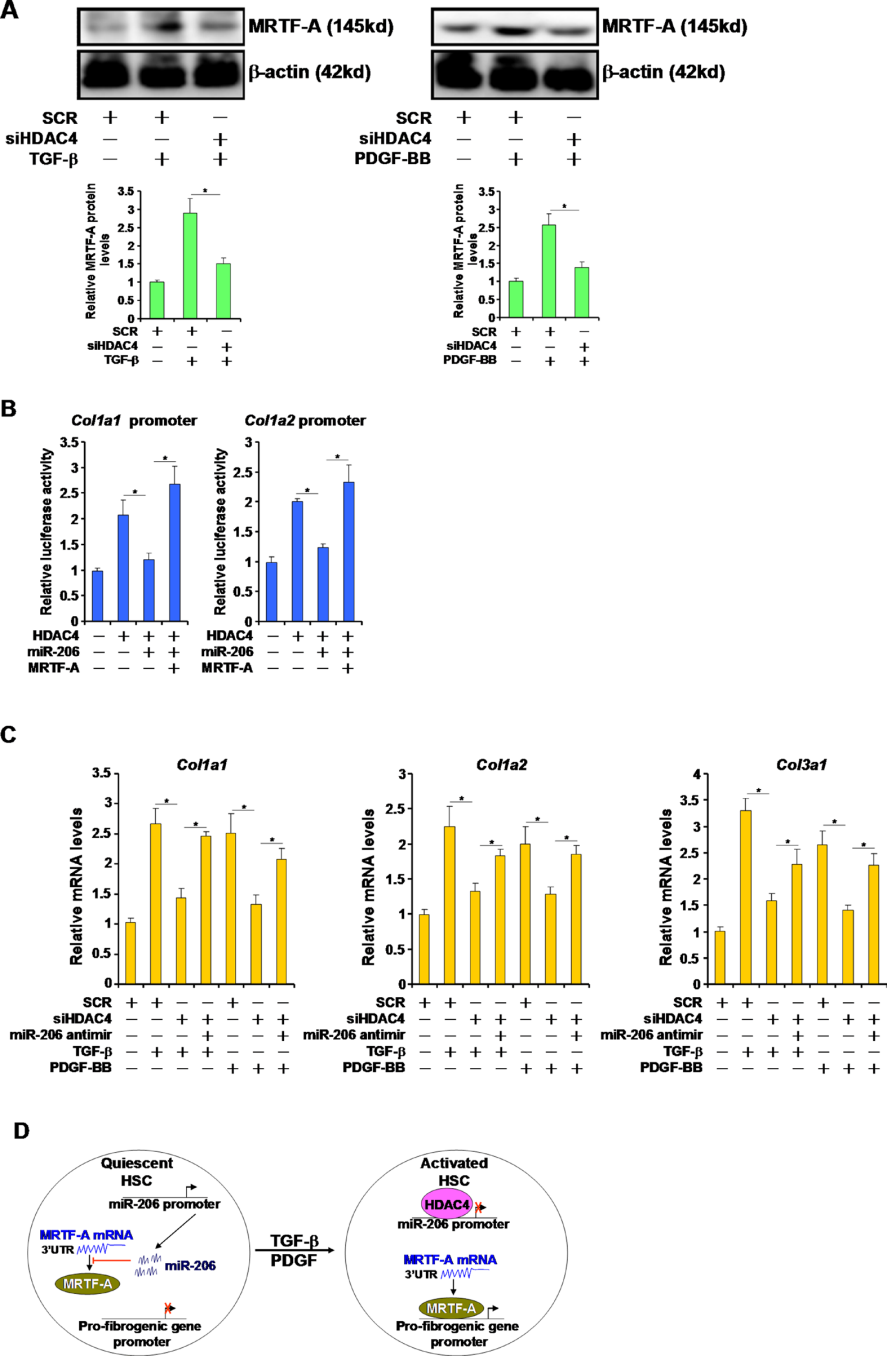
HDAC4 regulates fibrogenesis by targeting miR-206 (**A**) HSC-T6 cells were transfected with siRNA targeting HDAC4 or scrambled siRNA followed by treatment with TGF-β or PDGF-BB for 24 hours. Expression levels of MRTF-A were examined by Western. (**B**) Promoter-luciferase constructs were transfected into HSC-T6 cells with HDAC4, MRTF-A, and/or miR-206. Luciferase activities were normalized by both protein concentration and GFP fluorescence. (**C**) HSC-T6 cells were transfected with indicated siRNAs and/or miR-206 inhibitor followed by treatment with TGF-β or PDGF-BB for 24 hours. Expression levels of fibrogenic genes were examined by qPCR. (**D**) A schematic model. In quiescent HSCs, active transcription of miR-206 curbs MRTF-A protein levels and blocks trans-activation of pro-fibrogenic genes. In response to TGF-β or PDGF stimulation, HDAC4 is activated and turns off miR-206 transcription allowing MRTF-A proteins to accumulate. MRTF-A, in turn, turns on the transcription of pro-fibrogenic genes to promote HSC activation.

Since HDAC4 silencing blocked the induction of MRTF-A protein levels by TGF-β and PDGF (Figure 4A), we asked whether HDAC4 might regulate fibrogenesis by targeting miR-206. Over-expression of HDAC4 activated the promoter activities of key pro-fibrogenic genes, including *Col1a1* and *Col1a2*, in HSC-T6 cells (Figure 4B). This effect, however, was essentially neutralized by the introduction of miR-206 mimic and restored by MRTF-A over-expression (Figure 4B). On the other hand, qPCR analyses demonstrated that while HDAC4 knockdown attenuated the induction of endogenous levels of collagen type I and type III by TGF-β and PDGF, miR-206 antimir (inhibitor) reversed this trend and restored the expression of pro-fibrogenic genes (Figure 4C). Therefore, we propose that HDAC4-mediated suppression of miR-206 may play a role in liver fibrogenesis.

## DISCUSSION

We have previously shown MRTF-A plays a pivotal role in regulating HSC activation and liver fibrosis [4–6]. The present study highlights a novel mechanism whereby MRTF-A expression is regulated post-transcriptionally, via HDAC4-mediated repression of miR-206, during HSC activation and liver fibrosis in response to pro-fibrogenic stimuli. Several independent investigations have demonstrated divergent roles for HDAC4 in regulating liver pathologies. Barbier-Torres *et al* showed that HDAC4 promotes cholestatic liver injury whereas HDAC4 knockdown or inhibition alleviates liver fibrosis in mice [11]. Indeed, both Mannaerts *et al* and Huang *et al* have shown that HDAC4 contributes to HSC activation by repressing miR-29 in CCl4-induced [12] and BDL-induced [13] animal models of liver fibrosis. It is not entirely clear how miR-29 regulates HSC activation although it has been suggested that c-Fos, a key pro-fibrogenic transcription factor, could be a potential target for miR-29 [14]. Our data indicate that miR-206 is another downstream target of HDAC4 in the context of HSC activation and liver fibrosis and that miR-206 suppresses HSC activation likely through inhibiting MRTF-A expression. Therefore, we propose that HDAC4 may drive liver fibrogenesis via differentially regulating the expression of multiple miRNAs thereby simultaneously enhancing a string of pro-fibrogenic pathways. Of note, MRTF-A and c-Fos have been reported to form a complex and synergistically activate transcription of endothelin in vascular endothelial cells [15]. It remains to be determined whether MRTF-A and c-Fos, both downstream of HDAC4, could cooperate to activate pro-fibrogenic transcription in HSCs.

The data as presented here, however, should be interpreted with caution. First, HDAC4 might regulate HSC activation via alternative mechanisms. Fang *et al* have previously shown that HDAC4 represses SIRT1 transcription in skeletal muscle cells [16]. It is well documented that SIRT1 possesses anti-fibrogenic properties in both cultured cells and animal models [17–19]. Therefore, HDAC4 might stimulate HSC activation by alleviating SIRT1-dependent repression of fibrogenesis. Second, other potential targets of miR-206 may also contribute to HSC activation and liver fibrosis. Huang *et al*, for instance, have shown that miR-206 is involved in the fibrogenesis of skeletal muscle by inhibiting the expression of TGF-β [20]. miR-206 has also been shown to directly target SMAD3, key mediator of TGF-β induced fibrogenesis [21]. Of interest, the ability of MRTF-A to promote fibrogenic transcription seems to be controlled by TGF-β-induced nuclear translocation and rely on its interaction with SMAD3 [22]. It is possible that miR-206 may not only down-regulate MRTF-A expression but its activation as well to block HSC activation. Clearly these issues should be further investigated.

HDAC4 inhibition as a potential interventional strategy to treat liver diseases has been implicated in models of non-alcoholic steatosis and hepatocellular carcinoma [23, 24], both of which feature fibrosis as a key pathophysiological process. Our findings as summarized in this report reveal a novel mechanism whereby HDAC4 contributes to hepatic disorders. These data further validate the desirability of targeting HDAC4 to treat liver diseases.

## MATERIALS AND METHODS

### Animals

All animal protocols were reviewed and approved by the intramural Committee on Ethical and Humane Treatment of Experimental Animals of Nanjing Medical University. To induce liver fibrosis, 6–8 week-old male C57/BL mice were injected with CCl4 (1.0 mL/kg as 50% vol/vol) or thioacetamide (TAA, 100mg/kg) every other day for 7 days as previously described [4, 6]. Alternatively, the common bile duct of mice was ligated twice with silk sutures. Bile duct ligation (BDL) and sham-operated mice were sacrificed two weeks following the surgical procedure as previously described [5].

### Cell culture and treatment

Immortalized rat hepatic stellate cells (HSC-T6, ATCC) were maintained in DMEM supplemented with 10% fetal bovine serum (FBS, Invitrogen) and 1% penicillin-streptomycin. TGF-β (2ng/ml) and PDGF-BB (5ng/ml) were purchased from R&D.

### Plasmids, transfection, and reporter assay

HA-tagged HDAC4, the promoter-luciferase constructs for the *Col1a1* and *Col1a2* genes have been described previously [5, 16]. MRTF-A reporter plasmid was constructed by amplifying and fusing ~2kb of the 3′ un-translated region (UTR) of the MRTF-A mRNA to the pGL4 vector. Mutagenesis of the reporter plasmid was performed with the help of a Quickchange kit (Strategene). siRNA for Hdac4, 5′-GCAGAUCUGUGUUUUGAAA-3′. Transient transfections were performed with Lipofectamine 2000 (Invitrogen). Luciferase activities were assayed 24–48 hours after transfection using a luciferase reporter assay system (Promega). Experiments were routinely performed in triplicate wells and repeated three times.

### Protein extraction and Western

Liver tissues were homogenized using the MagNA Lyser instrument (Roche) and re-suspended in RIPA buffer as previously described [25]. The proteins were quantified with the BCA reagent (Pierce) according to the manufacturer's protocol, and separated by 10% polyacrylamide gel electrophoresis. Western blot analyses were performed with anti-MRTF-A (Santa Cruz), anti-HDAC4 (Abcam), anti-collagen type I (Rockland), anti-α-SMA, and anti-β-actin (Sigma).

### ChIP assay

ChIP assays were performed essentially as described previously [26–28]. Chromatin was cross-linked with 1% formaldehyde. Aliquots of lysates containing 200 μg of protein were used for each immunoprecipitation reaction with anti-HDAC4 (Abcam), anti-acetyl histone H3, and anti-acetyl histone H4 (Millipore), or pre-immune IgG. Precipitated genomic DNA was amplified by real-time PCR with the primers spanning the rat miR-206 proximal promoter region: Forward, TGCCAGTGTCCGTTCCTCTC, and Reverse, CTTAGAGCTTGCCAAGGAGCTTC.

### RNA extraction and real-time quantitative PCR

RNA was extracted using an RNeasy RNA isolation kit (Qiagen). Reverse transcriptase reactions were performed using a SuperScript First-strand synthesis system (Invitrogen). Real-time qPCR reactions were performed on an ABI STEPONE Plus (Life Tech).

### Statistical analysis

One-way ANOVA with post-hoc Scheffe analyses were performed using an SPSS package. *P* values smaller than .05 were considered statistically significant.
